# Association of social support and socio-demographic characteristics with poor self-rated health and depressive symptomatology among single mothers in Cyprus: a descriptive cross-sectional study

**DOI:** 10.1186/s12912-016-0134-x

**Published:** 2016-03-01

**Authors:** Elena Rousou, Christiana Kouta, Nicos Middleton

**Affiliations:** Nursing Department, Faculty of Health Sciences, Cyprus University of Technology, 15, Vragadinou str, 3041 Limassol, Cyprus

**Keywords:** Single parent family, Single mothers, Health, Depressive symptoms, Social support, Community nursing

## Abstract

**Background:**

The number of single-parent families headed by mothers is rapidly growing worldwide. A large part of the international literature reveals that single motherhood is associated with increased levels of chronic stress, mainly due to economic distress and reduced levels of social support, which may eventually lead to physical and psychological illness. Most published research comes from Northern Europe and the US, while it is accepted that both social welfare systems and societal factors vary substantially across countries. These issues haven’t been explored in Southern Europe and this study aims to fill this gap by a) assessing the health of single mothers in Cyprus in terms of self-assessed general health and the prevalence of clinical depressive symptoms and b) investigating the effect of perceived social support relation to their health status.

**Methods:**

General health was assessed in terms of Self-Rated Health (SRH), while the prevalence of clinical depressive symptoms was assessed with the Center of Epidemiological Studies-Depression Scale (CES-D). Perceived social Support was assessed with the Social Provision Scale. All scales were completed anonymously and voluntary by 316 single mothers. Univariable and multivariable associations between SRH and depressive symptoms with socio-demographic characteristics were investigated using chi-square tests and in multivariable backward stepwise logistic regression models respectively. Odd ratio of clinically significant depressive symptoms and SRH across decreasing levels of social support were estimated in logistic regression models.

**Results:**

The prevalence of depressive symptoms (CES-D score ≥ 22) was 38.9 %, which is almost three times greater than the general population. Strong associations with all health assessment tools were observed with variables relating to the lowest monthly family income and the presence of economic difficulties, unemployment, the single motherhood status and pre-existing illness. Social support as perceived by the mothers displayed a strong negative independent association with all tools, even after adjusting confounders.

**Conclusion:**

These findings can be a challenge for health care professionals, especially those working in the field of community-family nursing and highlight the necessity of interventions and strategies at community level in order to support this vulnerable population group.

## Background

The increasing number of single parent families has been one of the most significant structural changes in contemporary society, especially in the developed world [1]. In Europe, there has been a tremendous rise in divorce rates, from 0.9/1000 in 1970 to 2.0 in 2010 and births outside marriage, from 17.4 % in 1990 to 37.4 % in 2010 [2]. Despite a considerable increase in single fathers [3] and joint child custody arrangements in recent decades [4], the vast majority of single parent families are headed by a mother. In Cyprus, there has also been a significant rise in single mother households over the last decades, from 3.6 % in 1982, to 5.7 % in 2001 and 7.9 % in 2012 [2].

Recent studies suggest that family instability and dissolution and the transition to single parenthood influence parental well-being [5–7]. A series of circumstances common to single-parent families, such as increased daily stresses, task over-load, decreased financial resources, and reduced social support, may influence parenting adjustment and behavior. Women are more likely than men to be faced with the dual role of being a family’s sole caregiver and primary breadwinner and these factors may be consequential for women’s economic and social resources, as well as their psychological well-being [7]. At the same time, single parent households seems to be associated with adverse child outcomes and this may contribute to the intergenerational transmission of inequality [8, 9].

With regard to single mothers’ psychological health status, a wealth of published evidence exists [1, 5, 10]. Single mothers have been found to experience higher rates of psychological distress [11, 12], mood disorders [7] and anxiety disorders [13]. Depressive symptomatology has been found to be particularly prevalent among mothers in single parent families, commonly two to three times higher compared to the general population [10].

The majority of the studies examine the mental health consequences of single motherhood in relation to high levels of stress. By contrast, only few studies [14, 15] have focused on the role of social support, which is particularly important for the psychosocial wellbeing especially in subpopulations where stress is relatively high [16]. Mothers of young children, and especially single mothers, rely on family and friends for emotional as well as practical assistance in coping with the demands of children and daily stresses. Therefore, a perceived lack of support or dissatisfaction with the quality of the support may place the mother at risk of psychological distress [5].

Past literature examined the role of social support in relation to family structure (i.e., single mothers vs married counterparts), mainly in western societies and findings support that: 1) higher levels of social support is associated with lower levels of physical and mental health and 2) single mothers compared to their married counterparts experience lower levels of social support [14, 15]. The majorities of these studies though, focused on cross-cultural comparisons of different welfare regimes and policies [17], or investigated the impact on health caused by changes in economic or social circumstances [18]. Only a limited number of studies focused exclusively on single mothers, in order to investigate the association between depressive symptoms and social support [5, 15, 19].

Social support differs in cultural and socio-political context [17, 18]. Therefore, the socio-cultural context in Cyprus may not be ready to compromise with the rapid changes in family transition process and the subgroup of single mothers has difficulties to incorporate or to claim either their benefits or the support they really need. Despite that, there is complete lack of studies investigating the health of single mothers in Cyprus and possible associations with socio-demographic variables.

The aim of this study was to assess the self-rated health as well as estimate the prevalence of clinically depressive symptoms among single mothers in Cyprus. Furthermore, the study aimed to investigate possible associations with socio-demographic characteristics and the levels of perceived social supports.

## Methods

### Study population and design

A cross-sectional descriptive correlational study was conducted between January and March 2012, among single mothers (divorced, separated, widows or unmarried) who had the custody of at least one child under the age of 18. Single mothers with a longstanding affair or living with their parents were excluded. The study sample was recruited through the Single Mothers Association (SMA) (*N* = 179) as well as through snowball sampling to reach single mothers who were not members of the association (*N* = 136), in order to achieve a more heterogeneous sample in terms of their socio-demographic characteristics.

The questionnaire pack was distributed to single mothers, while they were visiting SMA. Each participant was asked to propose one or two single mothers they were acquainted with, who were not members of the SMA. 

### Instruments

#### The self-rated health (SRH)

We used a single self-rated health (SRH) question to assess the participants’ state of global health. The question posed was “How would you rate your general health?” using a five point scale ranging from “very good” to “very poor.” SRH is recommended as a health indicator by WHO [20], and is considered a good proxy of future morbidity and mortality [21, 22]. The SRH question has been used extensively in many populations, including single mothers.

#### The depression inventory of the center for epidemiology studies (CES-D)

The CES-D was developed by [23] to measure the severity of symptoms of depression in community populations, and has been used extensively as a screening tool. Items were selected to represent the major components of depression on the basis of the clinical literature and factor analytic studies. The scale is a composite of 20 items, four of which use positive wording to control for response bias. Subjects are asked to rate each item on a scale from 0 to 3 on the basis of ‘how often you have felt this way during the past week’, 0 = rarely or none of the time (less than 1 day), 1 = some or a little of the time (1–2 days), 2 = occasionally or a moderate amount of time (3–4 days), and 3 = most or all of the time (5–7 days). The CES-D score ranges from 0 to 60. Higher scores indicate more severe depressive symptoms [23].

The CES-D scale has been previously translated in Greek [24]. The reliability and validity of the CES-D have been tested in many populations. Internal consistency as measured by Cronbach’s alpha is high across a variety of populations; generally around 0.85 in community samples and 0.90 in patient samples [23]. In the current study, the Cronbach’s alpha coefficient among a pilot sample of *n* = 23 for internal consistency was 0.92. The average 3-week test-retest reliability coefficient for the CES-D total scores was 0.81.

#### Social provision scale

The Social Provision Scale, developed by Cutrona and Russel [25] based on the Social Provision Theory of Weiss (1974) [26]. It consists of 24 items with a 4-point scale ranging from “strongly disagree” to “strongly agree” and a theoretical range from 24 to 96. It assesses six provisions of social relationships described by Weiss [26], namely guidance (advice or information), reliable alliance (assurance that others can be counted on in times of stress), reassurance of worth (recognition of one’s competence), attachment (emotional closeness), social integration (a sense of belonging to a group of friends), and opportunity for nurturance (providing assistance to others). Half of the items are worded in a negative direction to control for response bias.

The SPS scale was translated from English to Greek by two independent translators, familiar with the Greek culture. The two versions of the instrument were compared in order to generate a single reconciled version, which was translated back into English [27]. The final version of the instrument was pre-tested with a group of 23 single mothers (pilot study) in order to assess the readability and general comprehensibility.

Using the Social Provisions Scale, scores can be derived for each of the six provisions as well as for a global social support score [25]. This scale has been used with a variety of samples [7, 28–30] that has supported the reliability and validity of the Social Provisions Scale, as well as the factor structure of the measure [25]. Scores on the measure have been shown to predict adaptation to stress among a wide variety of populations and individuals working in stressful job situations. Internal consistency of the total Social Provisions score was found to be .915 [25]. In the present study, the Cronbach’s alpha coefficient for internal consistency was 0.92.

### Ethical considerations

After a short briefing of the cover letter, participation in the study was voluntary and anonymous in order to guarantee confidentiality. Completed questionnaires were returned in sealed envelopes, and return of the questionnaire signified consent to take part. The study was approved by the National Bioethics committee (EEBK ΕΠ 2011.01.42), as well as the Ethics committee of the Cyprus University of Technology.

### Data analysis

Data were analyzed using SPSS 17.0. Descriptive statistics for all socio-demographic characteristics, subjective general health and depressive symptoms of the participants were calculated, expressed as appropriately in frequencies, mean values and standard deviation.

Concerning the SRH, no consensus exists as to whether self-rated health should be dichotomized, or whether the “fair (neither good nor bad health)” category should be regarded as “good health” when creating a dichotomous variable [15]. For the purpose of the analysis as well as to allow direct comparisons with prior studies that employed the same analysis used here [31]. Responses were classified into a binary variable in which “good” health included the responses “very good” and “good”, and “poor/fair” health included all other responses.

As for the CES-D scale, in primary care settings a cut-off value of 20–22 is commonly used, as this score increases its specificity of the instrument [23]. In the present study, scores of 22 or higher were identified as indicative of clinically significant depressive symptoms.

Quartiles of the total SPS score were used to characterize the level of perceived social support.

Univariable and multivariable associations between subjective general health and depressive symptoms with socio-demographic characteristics were investigated using chi-square tests and in multivariable backward stepwise logistic regression models respectively. Odd ratio (and 95 % confidence intervals) of clinically significant depressive symptoms and subjective general health across decreasing levels of social support were estimated in logistic regression models before and after adjusting for the potential confounding effect of socio-demographic variables. Fully adjusted model included all of the socio-demographics.

## Results

### The socio-demographic characteristics of the sample

The final sample consisted of 315 single mothers. Table 1 presents the socio-demographic characteristics of the participants. The mean age of the participants was 39.17 years (*SD* = 6.64, range: 23–59). More than half of the sample was between 35 and 44 years old. The vast majority (*n* = 231, 73.1 %) were divorced and only a small proportion were unmarried mothers (*n* = 25, 7.9 %). Nearly half of the sample were in a single mother family for more than 5 years (*n* = 147, 46.5 %). The majority had either one or two children (40.3 and 43.5 % respectively), and most of them had at least one child under the age of 12 years (*n* = 204, 64.9 %). More than half of the participants (58.9 %, *n* = 179) were members of the SMA.

**Table 1:** Socio-demographic characteristics of the participants and prevalence of clinically depressive symptoms (CES-D ≥ 22) and the level of SRH

		Less than good SRH				CES-D score > =22			
	% (*n*=)	%	*x* ^2^	Df	*P* value	%	*x* ^2^	Df	*P* value
Age									
23–34	25,4 (80)	31.2	4.942	2	0.085	38.8	0.406	2	0.816
35–44	51,7 (163)	43.6				40.5			
45–59	22,9 (72)	47.9				36.1			
Educational level									
Junior high school	7,9 (25)	60.0	4.33	3	0.228	52	12.02	3	0.007
High school	32,4 (102)	37.6				48			
Higher education	23.5 (74)	39.2				40.5			
University	36.2 (114)	42.1				27.2			
Single family status									
Separated	9,5 (30)	56.7	13.39	3	0.004	43.3	1.921	3	0.589
Widow	9,2 (29)	67.9				44.8			
Divorced	73,1 (231)	36.4				39			
Unmarried	7,9 (25)	40.0				28			
How long as single mother									
< 2 years	20.5 (65)	37.5	0.695	2	0.707	46.2	2.110	2	0.348
2–4 years	33 (103)	40.8				35.0			
5 or more years	46.5 (147)	43.5				38.8			
Children age < 12 years old									
Yes	64.9 (204)	37.3	4.416	1	0.036	40.2	0.256	1	0.613
No	35.1 (109)	49.2				37.3			
Employment status									
Economically inactive	8.2 (26)	53.8	5.99	3	0.112	57.7	8.818	3	0.032
Unemployed	7.6 (24)	20.8				37.5			
Working part-time	10.4 (33)	39.4				54.5			
Working full-time	73.4 (232)	42.4				34.9			
Monthly family income									
> 1000	27.8 (88)	42.0	4.77	5	0.444	54.5	16.16	5	0.006
1000–1499	27.2 (86)	36.0				38.4			
1500–1999	18.0 (57)	39.3				33.3			
2000–2499	10.1 (32)	37.5				34.4			
2500–3499	12.0 (38)	55.3				26.3			
≥ 3500	4.4 (14)	50.0				14.3			
Member of SMA									
No	56.8 (179)	43.0	0.522	1	0.490	36.3	1.184	1	0.296
Yes	43.2 (136)	39.0				42.3			
Single mothers’ allowance recipient									
Yes	58.9 (186)	38.9	1.14	1	0.285	43.5	3.866	1	0.049
No	40.8 (129)	45.0				32.6			
Financial child support from the father									
Yes	51.9 (163)	34.4	6.93	1	0.008	38	0.145	1	0.703
No	48.1 (152)	49.0				40.1			
Economic hardship during the last 12 months									
Yes	79.7 (252)	44.2	4.44	1	0.035	44	13.73	1	<0.001
No	20.3 (64)	29.7				18.8			
Limiting-long standing illness									
Yes	49.4 (156)	57.4	32.83	1	<0.001	52.6	24.12	1	<0.001
No	50.6 (160)	25.6				25.6			
Physical activity									
Yes	30.8 (97)	30.9	6.185	1	0.013	30.6	4.128	1	0.042
No	69.2 (218)	45.9				42.7			
Smoking habit									
Yes	39.4 (124)	41.1	0.002	1	0.967	37.1	0.287	1	0.637
No	60.6 (191)	41.1				40.1			
Alcohol consumption									
Yes	30.8 (97)	41.2	0.001	1	0.994	36.1	0.475	1	0.491
No	69.2 (218)	41.3				40.2			

Most single mothers had university education (*N* = 114, 36.2 %) and the vast majority held full-time jobs (*n* = 232, 73.4 %). Despite that, more than half of the participants (*n* = 174, 55.0 %) reported monthly family income less than €1500, and 27.8 % (*n* = 88) less than €1000. Apart from that, 40.8 % (*n* = 129) are not single mothers’ allowance recipients, and do not receive financial support from the father of their child/children. Not surprisingly, 79.7 % (*n* = 252) of the sample reported economic hardship to meet their daily expenses during the last 12 months.

Concerning their health status, nearly half of the sample (*n* = 156, 49.4 %) self-reported at least one long standing illness. Most commonly reported were depression (32.2 %), eczema (28 %) and thyroid gland disorders (24.6). Despite that, only 29.1 % of the sample reported that they have recently visited a doctor for that health problem.

Nearly half of the sample (41.3 %, *n* = 130) rated their level of general health as poor/fair. Additionally, 38.9 % (*n* = 123) scored 22 or higher in the CES-D scale, indicating clinically depressive symptoms.

### Prevalence of poor/fair self-rated general health and clinically depressive symptoms in relation to socio-demographic characteristics

Among single mothers who reported economic hardship for daily expenses in the last 12 months, both the prevalence of poor self-rated health (44.2 vs 29.7 %, x^2^ = 4.44, df = 1, *p* = 0.035), as well as the prevalence of clinically depressive symptoms (44.0 vs 18.8 %, x^2^ = 13.73, df = 1, *p* = <0.001) appeared much higher than those who did not report economic hardship. Similarly, statistically significant higher prevalence of poor self-rated health and clinically depressive symptoms were observed among those reporting long standing illness. (SRH: 57.4 vs 25.6 %, x^2^ = 32.83, df =1, *p* = <0.001 – CES-D: 52.6 vs 25.6 %, x^2^ = 24.12, df =1, *p* = <0.001).

However, there have been some variations concerning the factors associated with SRH and those associated with the prevalence of clinically depressive symptoms. As for the type of single motherhood, even though no associations were observed with depressive symptoms, widows and separated single mothers were more likely to report their subjective state of general health poor/fair (67.95 and 56.7 % respectively) compared to unmarried mothers (40 %) (x^2^ = 13.39, df = 3, *p* = 0.004).

Additionally, statistical significance with lower levels of SRH was observed among those who had more than 3 children (*p* = 0.036), had children younger than 12 years (*p* = 0.036) and among those who do not get financial support from the father of their children (49 vs 34.3 %,%, x^2^ = 6.93, df = 1, *p* = 0.008). Furthermore, even though no statistical significance has been found, the prevalence of poor/fair subjective general health tended to be higher with increasing age and lower with higher level of education (Table 1).

The prevalence of clinically significant depressive symptoms was statistically significantly higher among single mothers who had junior high school education compared to those who had university education (52 vs 27.2 %, x^2^ = 12.02, df = 3, *p* = 0.007), and among single mothers who were economically inactive (57.7 %, x^2^ = 8.81, df = 3, *p* = 0.032). Additionally, single mothers who were recipients of single mothers’ allowance (x^2^ = 3.86, df = 1, *p* = 0.049) and reported lower monthly family income (x^2^ = 16.16, df = 5, *p* = 0.006) were more likely to experience clinically depressive symptoms.

The results of the backward stepwise logistic regression analysis are presented in Tables 2 and 3. The strongest associations with SRH were observed with single motherhood status, with widows more likely to report worse SRH (OR 3.3, 95 % CI: 1.01–10.7). In addition, single mothers who were unemployed were less likely to report worse their self-rated level of health. Independent associations in the magnitude of 1.7–2.1-fold increase in the odds of reporting worse SRH were also observed among those who were not receiving financial support from the father of their chil/dren (OR 1.79, 95 % CI: 1.05–2.93) and among those not physically active (OR 2.11, 95 % CI: 1.22–3.65).

**Table 2:** Adjusted odds ratios (and 95 % CI) of worse SRH or prevalence of clinical depressive symptoms (CES-D ≥ 22) by personal, family, economic and health behavior characteristics as estimated in multivariable backward stepwise logistic regression analysis

	SRH less than good		CES-D (≥22)	
	Adjusted^a^	*P* value	Adjusted^a^	*P* value
	OR (95 % CI)		OR (95 % CI)	
Single motherhood status				
Unmarried	1	-----	1	-------
Divorced	1,084 (0,438–2,681)	0,862	2.051 (0.778–5.406)	0,146
Separated	2,640 (0,831–8,388)	0,100	4.308 (1.224–15.170)	0,023
Widow	3,300 (1,016–10,717)	0,047	3.247 (0.960–10.982)	0,058
Employment status				
Full time	1	-----		
Part time	0,711 (0,321–1,577)	0,402		
Unemployed	0,187 (0,059–0,592)	0,004		
Not working	1,268 (0,534–3,016)	0,590		
Single motherhood allowance recipient				
No			1	-------
Yes			5.825 (1.195–28.399)	0,029
Financial child support from the father				
Yes	1	-----		
No	1,759 (1,056–2,931)	0,030		
Physical activity				
Yes	1	-----	1	-------
No	2,119 (1,228–3,655)	0,007	1,587 (0,933–2,702)	0,089

^a^Factors included in the first stage: age, educational level, employment status, single motherhood status, duration as single mother, number and age of independent child/ren, monthly family income, single mothers’ allowance, financial support from the father, member of the Single mothers’ association, smoking habit, alcohol consumption, physical activity

**Table 3:** Adjusted odd ratios (and 95 % CI) for the association between SRH or CES-D scores with decreasing level of Social Provision (SPS) as estimated in step-wise four-level multivariate analysis

	Prevalence of poor/fair health (SRH)	Unadjusted	Fully Adjusted^a^	Prevalence of Depressive Symptoms (CES-D score ≥22)	Unadjusted	Fully Adjusted^a^
			Model IV			Model IV
		OR (95 % CI)	OR (95 % CI)		OR (95 % CI)	OR (95 % CI)
		*P* value	*P* value		*P* value	*P* value
T4: Higher quartile of perceived social support (values: 87–96)	33,3 %	1	1	11.7 %	1	1
Τ3	36,0 %	0.944	0.797	28.9 %	2.008	1,846
(values:78–86)		(0,493–1.804)	(0.341–1.864)		(0,940–4.288)	(0,726–4.696)
		0,861	0,600		0,072	0,198
Τ2	30,1 %	0.749	0.573	34.0 %	2,749	3,828
(values:71–77)		(0.392–1.432)	(0.245–1.342)		(1.321–5.717)	(1,520–9.644)
		0,382	0,200		0,007	0,004
Τ1: Lower quartile of perceived social support	63,5 %	2.920	2.758	78.4 %	17,866	20.523
		(1.525–5.590)	(1.125–6.761)		(8.046–39.672)	(7.575–55.601)
(values:42–70)		0,001	0,027		0,000	0,000

^a^Variables included: single family type, monthly family income, single mother allowance recipient, financial support from the father, member of SMA, health related behaviors, age, educational status, number and age of children, employment status, economic difficulties, presence of long standing illness

The strongest associations with the CES-D were observed with single motherhood status, with those separated more likely to report clinical depressive symptoms (OR 4.3, 95 % CI: 1.22–15.17), among those who were not receiving single mothers’ allowance (OR 5.8, 95 % CI: 1.19–28.39), and those who were not exercising (OR 1.5, 95 % CI: 0.93–2.7). Results were unchanged when using forward method instead.

### Perceived social support among single mothers and association with health outcomes

The minimum and maximum SPS observed scores were 42 and 96 respectively (theoretical range: 24–96). The socio-demographic characteristics that were statistically significantly related with lower perceived levels of social support were: lower monthly family income (*p* = 0.001), experiencing economic difficulties to meet daily expenses (*p* = 0.001), being a recipient of single mother allowance (*p* = 0.001), unmarried mothers vs divorced, separated or widows (*p* = 0.007) and not registered in the Single Mothers’ association (*p* = 0.009) – results not shown in detail.

Table 2 presents the associations of SRH and CES-D scores with decreasing levels of SPS before and after adjusting for all socio-demographic variables which showed an association with either the health outcomes and/or social support.

The prevalence of poor/fair level of general health, was nearly triple (OR 2.92, 95 % CI: 1.52–5.59) among single mothers reporting the lower levels of social support compared to those in the higher quartile. After adjusting for the potential confounding effect of socio-demographic variables, the association still persisted and no significant differences were observed (OR = 2.75 95 % CI: 1.12–6.76).

An even stronger association with perceived level of social support was observed with the clinically significant depressive symptoms. In fact, unlike self-rated health where the observed association appeared restricted to the quartile of mothers with the lowest SPS scores, in the case of CES-D a stepwise increase was observed with decreasing levels of SPS. Single mothers who experience the lower level of social support were nearly 18 times more likely to suffer depressive symptoms (OR = 17.88 95 % CI: 8.04–39.67). This association not only didn’t attenuate in the fully adjusted model, but it appeared even stronger (OR = 20.52 95 % CI: 7.57–55.60).

## Discussion

### Main findings

As many as 41.3 % of single mothers in this study assessed their level of health as poor/fair. SRH is a good predictor of mortality and morbidity [22], and thus the high prevalence of SRH estimated in this study might be indicative of poor health among single mothers in Cyprus. The majority of studies in the literature indicated that single mothers generally tend to assess their level of health as lower compared to partnered mothers [32]. This estimate is comparable to those observed in Italy and Great Britain [17], slightly greater than in Finland and Sweden [18, 33] where welfare regimes seems to support single mothers but much lower than in South Korea [15]. As high as 60 % of single mothers compared to 28.7 % of their partnered counterparts in South Korea assessed their level of health as poor/fair and this inequality was mostly associated with economic hardship and reduced levels of social support.

Furthermore, 38.9 % of single mothers in Cyprus report clinically depressive symptoms, which is nearly three times higher than the lifetime prevalence of depression among the general population according to Eurostat [34]. This estimate is also consistent with previous studies [5, 7, 35], and is of particular concern since mental health is strongly associated with physical health [36].

Moreover, mental health is an important health outcome in itself and the high prevalence of depressive symptoms among single mothers in Cyprus must be taken into serious consideration, both for the well-being of this vulnerable population group as well as their children since maternal psychiatric disturbances and various problems in children are well established [37].

### Socio-demographic correlates with poor/fair Self-rated health and the presence of clinically depressive symptoms

In contrast to previous published studies [5, 12, 13, 38], participants in this study have higher educational attainment and a large proportion held a full time job. On the other hand, the findings are consistent with the majority of the studies who report that single mothers have lower income and thus receive welfare allowances, and experience financial hardship with daily expenses [17, 32, 39].

The strongest and most consistent associations with both lower self-reported level of health and clinically depressive symptoms were financial hardship and the presence of a longstanding illness. These findings are also consistent with the majority of the relevant studies [40]. Significant associations were also observed with either depressive symptoms or worse self-assessed level of health and educational level, employment status, family income, single family status and children age [38, 41, 42].

### Economic means and financial hardship

The present study adds more evidence to the existing literature with regards to the adverse association of economic hardship with health status among single mothers. Single mothers who had the lowest educational attainment, who were economically inactive, and consequently had lower monthly family income, were most likely to report worse health outcomes [7, 35, 39].

Unsurprisingly, in this study a statistically significant association was observed between monthly family income and health. This finding is consistent with the vast majority of previous studies [16, 43].

Furthermore, employment status was strongly related with SRH. Single mothers who did not work were more likely to assess their level of health lower (OR = 1.26). On the other hand, in this study single mothers who hold a part-time job were more likely to experience clinical depressive symptoms compared to those who were working full time. Even though, the cross-sectional nature of this study, does not allow to infer causality in the observed relationship between employment status and health, it should be noted that there is a complexity in this relationship.

First of all, several factors seem to affect the ability of the single-parent households’ primary earner to work. These factors include the employment opportunities offered by the labor market, the adequate social infrastructure, the availability of part time jobs, the flexibility of the working schedules, the social and welfare settings, and the level of tangible support from the extended family [44].

On the one hand, work increases monthly family income and restricts economic difficulties thus reduces the related stress [16]. In addition, socialization through work is considered a protective factor for health [44]. However, related data shows that even though single mothers are more likely to be employed than any other group of women [39, 44], they hold positions with lower earnings, or by necessity, are working part-time as they do not have the necessary support for the care of their children. This is strongly evident in the present study, since despite the fact that the great majority of the sample has high educational level (55.9 % higher education) and work full-time (73.7 %), the majority reported their monthly family income less than 1500 euro (53.1 %). Despite that, 40.8 % of single mothers are not beneficiaries for the single motherhood allowance, a finding which may reflect deficiencies in the Cyprus social welfare system.

On the other hand, single mothers who hold full-time jobs are more likely to experience increased level of stress, apparently as it is more difficult to combine work and family life, mainly due to the lack of supporting arrangements [12].

### Differences in relation to personal and family characteristics

Regarding personal characteristic, age was more strongly and positively related with the SRH and this is supported by several longitudinal studies [45, 46]. Single mothers had increased chances to evaluate their health lower at the 40–50 years age group compared to women in all other younger sub-groups. Only one study was found which contradict this finding [47] in Sweden, where single mothers in the younger age group (16–24 years) were more likely to report their level of health worse than the other sub-groups.

In contrast, even though not statistically significant, younger women were more likely to report in a higher frequency, clinical depressive symptoms, in line with several related studies [5, 35].

Generally, the differences observed between the age groups have been attributed to a number of related factors. Age is positively related with long standing illnesses and therefore more likely to report SRH as worse [45, 46]. On the other hand, younger single mothers are more likely to have been single parents for shorter period of time [48, 49], or have children less than 12 years old [50, 51], factors that have been shown to be strongly related with higher frequency of clinical depressive symptoms in the present study as well.

Moreover, our study showed that the type of single motherhood is strongly related with both SRH and clinical depressive symptoms. The dissolution of a marriage, whether due to divorce or widowhood, generally appears to significantly increase the likelihood of mental problems, such as anxiety disorder and depression [48], increase the levels of psychological distress [49, 52] and reduce the sense of mental wellness [53, 54]. In the present study, widowhood was strongly related with SRH (OR 3.3 95 % CI: 1.01–10.71) while separated single mothers reported a higher frequency of clinical depressive symptoms (OR 4.3 95 % CI: 1.22–15.17).

It can be assumed that the worse SRH among widows might be confounded by age, while the higher frequency of clinical depressive symptoms among separated mothers could be mainly due to the increase of stressors followed by the dissolution of a relation/marriage.

### Differences in relation to health related behaviors

An additional risk factor revealed was that unlike smoking or drinking habit, physical exercise was strongly and independently related with the health outcome of the sample. Despite the well-established beneficial effect of physical exercise in health [55], this finding should be integrated with caution due to the cross-sectional nature of the study.

However, the socio-economic characteristics of this population group as well as the reverse causality of this association must be taken into account. Single mothers who spent time for physical activity, possibly are financially well-off in order to afford it, they have the time required or the necessary tangible support to do so. However, this study highlights the potential for using physical activity as a possible health screening question.

### Association of perceived social support and health outcomes

The findings in this study indicated inverse relationship between depression and social support suggesting that if perceived social support is strong then depression will be weaker. These findings are consistent with past researches [25, 56, 57]. Malik [56] pointed out that social support and depression are not identical constructs. Depression and social support are two independent but related phenomenons. The emotional support provided by social ties enhances psychological well-being, which, in turn, may reduce the risk of unhealthy behaviors and poor physical health [58, 59]. Findings explained that the consequences of decrease in social support results in depression whereas the likelihood of experiencing depression can be decreased by supportive relationships.

## Limitations

The main limitation of this study is that the data were cross-sectional in nature. However the cross-sectional nature of this study precludes conclusions about the direction of the observed relationships between social support and health related behaviors with poor health due to reverse causality. This issue should be further explored in longitudinal studies. In addition, the data set did not include some measures known to be significantly associated with poor mental and physical health in single mothers, e.g., quality of relationship or experience of violence prior to divorce/separation [60]. Most critically, we could not assess the extent to which the measures available may have been markers of more entrenched and long-term disadvantage.

Despite limitations aforementioned, this study is the first to describe the health status of single mothers and its association with socio-demographic factors as well as level of perceived social support, which may, to a great extent, highlights the uneven distribution of socioeconomic resources and sub-optimal social welfare in Cyprus.

## Implications for practice

Understanding the causal mechanisms of mental health problems among single mothers is important for informing policy development. While the cross-sectional nature of the data limits the extent to which conclusions about causation can be made, findings suggest some possible policy implications. Given the importance of financial hardship, an effective policy solution may be to target solution oriented coping strategies to single mothers, such as financial management interventions in an effort to reduce the potential risk of poor health among mothers and their children.

Additionally, in order to be effective, policies and interventions must account for the ways in which social constraints and resources influence health across social groups. Solid scientific evidence establishing the causal impact of social ties on health provides the impetus for policy makers to ensure that health policy works to protect and promote social ties that benefit health. Moreover, care must be taken to develop strategies that increase the power of social ties to enhance individual health without imposing additional strains on care providers. The most socially isolated persons are those at greatest risk of poor health and early mortality [61].

The identification and treatment of mental health problems is clearly important for the success of welfare reform. In addition to being a barrier to self-sufficiency, psychiatric illness has important implications for more general social and family functioning. Improving the home environments of young children is a central, though occasionally overlooked, policy goal. The psychiatric disorder prevalence rates we document have important implications for child well-being. High levels of depression, stress, and other mental health problems constitute important barriers to effective parenting [62], and children of depressed mothers are at a greater risk for adjustment problems [63]. Therefore, improving mothers’ mental health will benefit child outcomes through improvements in parenting skills [64] and community as a whole.

## Conclusion

An alarming prevalence of poor self-assessed level of health and depressive symptoms was observed among single mothers in Cyprus. Furthermore, a strong and independent association between social support and single mothers’ health was observed.

Notably, some groups, and in this case single mothers, are more likely than others to experience social isolation or have greater risk for illness and disease than others. This group should receive higher priority in policy efforts and existing policies should also be re-evaluated. Therefore, coordinated programs could help identify socially isolated single mothers, and they could mobilize local resources to offer social and instrumental support to these individuals.
